# A Multifunctional Drug Combination Shows Highly Potent Therapeutic Efficacy against Human Cancer Xenografts in Athymic Mice

**DOI:** 10.1371/journal.pone.0115790

**Published:** 2014-12-22

**Authors:** Xiu-Jun Liu, Yan-Bo Zheng, Yi Li, Shu-Ying Wu, Yong-Su Zhen

**Affiliations:** Institute of Medicinal Biotechnology, Chinese Academy of Medical Sciences and Peking Union Medical College, Beijing, China; Rajiv Gandhi Centre for Biotechnology, India

## Abstract

The tumor microenvironment plays a crucial role during tumor development. Integrated combination of drugs that target tumor microenvironment is a promising approach to anticancer therapy. Here, we report a multifunctional combination of low-cytotoxic drugs composed of dipyridamole, bestatin and dexamethasone (DBDx) which mainly acts on the tumor microenvironment shows highly potent antitumor efficacy *in vivo*. In mouse hepatoma H22 model, the triple drug combination showed synergistic and highly potent antitumor efficacy. The combination indices of various combinations of the triple drugs were between 0.2 and 0.5. DBDx inhibited the growth of a panel of human tumor xenografts and showed no obvious systemic toxicity. At tolerated doses, DBDx suppressed the growth of human hepatocellular carcinoma BEL-7402, HepG2, and lung adenocarcinoma A549 xenografts by 94.5%, 93.7% and 96.9%, respectively. Clonogenic assay demonstrated that DBDx showed weak cytotoxicity. Western blot showed that Flk1 and Nos3 were down-regulated in the DBDx-treated group. Proteomic analysis showed that DBDx mainly affected the metabolic process and immune system process; in addition, the angiogenesis and VEGF signaling pathway were also affected. Conclusively, DBDx, a multifunctional drug combination of three low-cytotoxic drugs, shows synergistic and highly potent antitumor efficacy evidently mediated by the modulation of tumor microenvironment. Based on its low-cytotoxic attributes and its broad-spectrum antitumor therapeutic efficacy, this multifunctional combination might be useful in the treatment of cancers, especially those refractory to conventional chemotherapeutics.

## Introduction

Cancer is a complex disease involving the changes of tumor cells and the tumor microenvironment [1]. As reported, the tumor microenvironment changes in association with tumor development and promotes tumor growth and metastasis [2], [3]. Uses of drugs that target the tumor microenvironment provide a promising strategy for cancer therapy [4]–[5]. The combination of drugs that target the tumor microenvironment has been proved to be effective in cancer treatment [6]–[8]. Here, we report a multifunctional drug combination composed of dipyridamole (DPM), bestatin (BEN) and dexamethasone (DEX) which mainly targets the tumor microenvironment and its highly potent therapeutic efficacy.

Dipyridamole, a well-known anti-thrombotic drug, is an active nucleoside transport inhibitor. It can enhance the antitumor activity of many antimetabolites, such as 5-fluorouracil and methotrexate [9]. Dipyridamole can also impair tumor microenvironment and prevent breast-cancer progression in mice [10]. Bestatin (Ubenimex), an aminopeptidase inhibitor, has shown diverse antitumor activities and immunomodulatory activities [11], [12]. Clinically, bestatin is used as an immunomodulator in combination with chemotherapy or radiotherapy [13]. Bestatin can inhibit tumor cell proliferation and suppress tumor angiogenesis [14], [15]. Dexamethasone is a widely used drug of the glucocorticoid steroid family with potent anti-inflammatory and immunosuppressant effects. In clinics, it is often used to treat inflammatory and autoimmune diseases. In tumor treatment, dexamethasone is generally used for alleviate the side effects of chemotherapy [16]. There are reports that dexamethasone can also suppress tumor angiogenesis [17], [18].

In this study, we designed an integrated, multifunctional combination including dipyridamole, bestatin and dexamethasone and investigated its antitumor activity, particularly its therapeutic efficacy *in vivo*. Our research proves that DBDx is a highly effective, broad-spectrum antitumor combination predominantly targeting the tumor microenvironment.

## Materials and Methods

### Materials

Dipyridamole and dexamethasone were obtained from National Institutes for Food and Drug Control (China). Bestatin was provided by Apeloa Kangyu (China). For double or triple combinations preparation, the agents were mixed according to the indicated doses in saline, then ground and homogenized by using a mortar. Gemcitabine (Gemzar) was purchased from Lilly, France. Gefitinib (IRESSA) was from AstraZeneca. 5-FU was from Shanghai Xudong Haipu Pharmaceutical Co., Ltd. All chemicals and biochemical agents used were of analytical grade.

### Cell culture

Human hepatocellular carcinoma BEL-7402 cells were obtained from Shanghai Institute of Cell Biology, Chinese Academy of Sciences. Human hepatocellular carcinoma HepG2 cells were purchased from ATCC. Human lung adenocarcinoma A549 cells and human epidermoid carcinoma A431 cells were obtained from the Cell Center of the Institute of Basic Medical Sciences, Chinese Academy of Medical Sciences and Peking Union Medical College. Human pulmonary giant cell carcinoma PG cells were obtained from Department of Pathology, Peking University Health Science Center. All of these cell lines were cryopreserved in our laboratory and cultured at 37°C in RPMI-1640 medium (Gibco BRL Inc.) supplemented with 10% fetal bovine serum (Gibco BRL Inc.), 2 mM glutamine, 100 µg/mL streptomycin, and 100 U/mL penicillin in a humidified atmosphere containing 5% CO_2_.

### In vivo therapy study

All Kunming mice and NIH (nu/nu) athymic mice were purchased from Vital River Laboratories (Beijing, China).

In mouse hepatoma 22 (H22) model, female Kunming mice (18–22 g) were randomly divided with 10 mice for each group. On day 0, murine hepatoma 22 cells from ascites of tumor-bearing mice were transplanted subcutaneously into the right axilla region with 1.5×10^6^ cells/mouse. From day 3 to day 12, the tumor-bearing mice were treated orally with saline, single agents, double or triple combinations, respectively. 5-FU was administrated with the same schedule but given intraperitoneally. At day 14 all mice were sacrificed. Tumor weights were measured and the inhibition rates of tumor growth were calculated. Combination index (CI) was analyzed according to the Chou and Talalay method [19].

In hepatoma BEL-7402 xenograft model, human hepatoma BEL-7402 cells (1×10^7^) suspended in 200 µL saline were inoculated subcutaneously (s.c.) in the right armpit of female NIH *(nu/nu)* athymic mice (18–22 g). After 3 wk, the tumors were dissected and pieces of tumor tissue (2 mm^3^ in size) were transplanted s.c. by a trocar into athymic mice. When the tumor size reached about 100 mm^3^ (day 7), mice were divided with 6 mice per group and treated orally with saline or the drug combinations at different doses respectively, once daily, 5 consecutive days a week for 2 weeks. Tumor size and body weight were measured every 3–4 days. Tumor volume was calculated with the formula: V = ab^2^/2, where *a* represents the longitudinal diameter and *b* the perpendicular diameter. The inhibition rates of tumor growth were calculated according to the tumor volume.

For hepatoma HepG2 xenograft model, lung carcinoma A549 xenograft model, lung carcinoma PG xenograft model and epidermoid carcinoma A431 xenograft model, tumors were inoculated in athymic mice as described in BEL-7402 model. Schedules of drug administration were described in the results.

### Clonogenic assay

BEL-7402 cells of exponential growth were seeded at 50 cells per well in 96-well plates and cultured for 24 h at 37°C. Then various concentrations of drugs were added in triplicate and the cells were cultured for another 7 days. Colonies of greater than 50 cells were counted. The survival fractions were calculated according to the following formula: survival fraction (%)  =  counts of test wells/counts of control well×100%.

### Acute toxicity test

Kunming mice (18–22 g, half male and half female) were randomly divided into 4 groups with 20 mice per group. DBDx (ratio of dipyridamole, bestatin, and dexamethasone was 100, 20, and 1) was given orally at a single dose of 0, 1.28, 1.6 and 2 g/kg, respectively. Body weight of the mice, neurologic response, and behavior abnormality were closely monitored for 14 days.

### Western blot analysis

In H22 model, 3 days after tumor implantation, DBDx at 242 mg/kg were given orally, once daily, for 10 days. At day 14, the tumor tissues were isolated, 5 tissue specimens were taken from each group. The tumor tissues were lysed in the lysis buffer (50 mM Tris-HCl, 150 mM NaCl, 0.1% SDS, 1% NP-40, 0.5% Sodium deoxycholate, 100 µg/mL phenylmethylsulfonyl fluoride (PMSF), 1 µg/mL aprotinin, pH 8.0) and homogenized with a handheld homogenizer. The lysates were centrifuged at 12 000 rpm for 10 min and the supernatants containing protein were quantified using a BCA protein assay kit (Pierce).

The protein samples were electrophoresed on 10% SDS-PAGE, and transferred to PVDF membrane. After blocked with 1% BSA, the membrane was incubated with primary antibody (Santa Cruz) and HRP-conjugated secondary antibody (Zhongshan Inc.), sequentially. The immunoreactive band was visualized using Western blot luminol reagent (Santa Cruz Biotechnology, Inc.), and the image was captured using image analysis system (AIO Inc.).

### Sample preparation for two-dimensional differential gel electrophoresis

BALB/c athymic mice bearing BEL-7402 xenografts were divided into two groups, 5 mice for each group. One group of mice was treated with 242 mg/kg DBDx, once a day for 10 days; another group of mice was administered with saline as control. Next day after the last administration mice were sacrificed. Tumor tissues were collected and snap frozen with liquid nitrogen and stored at −80°C until processed.

Protein sample preparation, two-dimensional electrophoresis (2-DE) and mass spectrometry (MS) were performed at Beijing Protein Innovation.

Protein samples were prepared using trichloroacetic acid (TCA)-acetone precipitation method. Briefly, about 50 mg of tumor tissue frozen in liquid nitrogen previously were crushed by a metal mortar, and then suspended with 10% TCA in acetone containing 1 mM PMSF, 2 mM ethylenediamine tetraacetic acid (EDTA) and 10 mM dithiothreitol (DTT) for 2 h. After centrifugation, the precipitated protein was washed with precooled acetone. The pellet was dissolved in the lysis buffer containing 20 mM Tris-HCl, pH 7.5, 8 M urea, 4% 3-[(3-cholamidopropyl) dimethylammonio]- 1-propanesulfonate (CHAPS), 0.5% Pharmalyte (pH 3–10L), 10 mM DTT, 1 mM PMSF, and 2 mM EDTA. The lysate was sonicated for 5 min followed by centrifugation at 40 000 g for 15 min. The supernatant protein was quantified using Bradford method. The “mixed protein pools” were prepared by mixing equal amounts of protein from 5 tumor samples of the same group for 2-DE.

### 2-DE and image analysis

The “mixed protein samples” (200 µg) were mixed with rehydration buffer and applied to an 18 cm linear IPG strips, pH 3–10 (Amersham Biosciences, Sweden).Then the strips were subjected to isoelectric focusing in IPGphor (Amersham Biosciences, Sweden). The focused strips were subsequently reduced with 1% DTT and alkylated with 2.5% iodoacetamide (IAM) in equilibrated buffer (6 M urea, 30% glycerol, 2% SDS, and 50 mM Tris-HCl, pH 8.8). The treated strips were transferred to 12% SDS-PAGE in Ettan DALT II System (Amersham Biosciences, Sweden) for the secondary electrophoresis. The analytical gels were stained with silver staining without addition of glutaraldehyde. The stained gels were scanned using an Imagescanner (Amersham Biosciences, Sweden), and the images were analyzed using ImageMaster 2-D Platinum version 3.0 (Amersham Biosciences, Sweden).

### MS and protein identification

The differential expression spots were excised from gels and placed in Eppendorf tubes. The gel pieces were reduced with 10 mM DTT and alkylated with 55 mM IAM. Then the gels were sequentially equilibrated with 25 mM ammonium bicarbonate, 50% acetonitrile (ACN) +25 mM ammonium bicarbonate and 100% ACN for 10 min, followed desiccated in a vacuum centrifuge for 10 min. The dried gels were rehydrated in digestion solution (0.01 µg/mL trypsin dissolved in 25 mM ammonium bicarbonate) on ice for 30 min, and incubated in 25 mM ammonium bicarbonate (10–15 µL) overnight at 37°C. The digestion was stopped using 0.1% triflouroacetic acid (TFA). The digested peptides were spotted onto the target (AnchorChip™, Bruker, Germany) and co-crystallized with α-Cyano-4-hydroxycinnamic acid (CHCA, 4 mg/mL in 70%ACN and 0.1%TFA). Then the dried matrices were analyzed by an Ultraflex MALDI-TOF/TOFII MS (Bruker, Germany) operated in the reflector mode in the m/z range from 600 to 4 000. Calibration for PMF (peptide mass fingerprinting) samples was performed externally using a mixture of standard peptides and internally using the peptide fragments of trypsin autolysis products. According to the PMF signals, the top three highest peptides with higher accuracy and higher abundance were further analyzed in the MS/MS mode. PMFs were analyzed with MASCOT (Matrix Science, U.K.) against NCBInr database. A mass accuracy tolerance was allowed within 100 ppm. Protein identifications were performed based on probability-based Mowse scoring algorithm with a confidence level of 95%.Then the identified proteins were analyzed using PANTHER (Protein ANalysis THrough Evolutionary Relationships) classification system (www.pantherdb.org).The system was designed to classify proteins (and their genes) and categorizes them according to family and subfamily, molecular function, biological processes, and pathways.

### Ethics Statement

All animal experiments were approved by the Ethics Committee for Animal Experiments of the Institute of Medicinal Biotechnology, Chinese Academy of Medical Sciences (IMBF20060302). The study protocols comply with the recommendations in the Regulation for the Management of Laboratory Animals of the Ministry of Science and Technology of China.

## Results

### Triple combinations showed improved and synergistic antitumor efficacy

The antitumor efficacy of triple combinations composed of DPM, BEN and DEX was first compared with that of single agents or double combinations in mouse hepatoma H22 model. After 3 days of tumor implantation (tumor volume: 250±30 mm^3^), drugs were given orally, once daily, for 10 days. At day 14, the mice were sacrificed and the tumor weight was measured. At the indicated doses in Table 1, the inhibition rates of respective single agents ranged from 22.8% to 68.6%. For the double combinations, the inhibition rates were from 58.7% to 66.3%. The CI values of double combinations were 0.8-1, indicated additive or slightly synergistic effects. For the triple combinations, 3×3 groups of combinations were studied as showed in Table 1. When three drugs combined together, the inhibition rates climbed up to 79.2%–89.4%, which were statistically different from that of relevant single agents or double combinations. The CI values of all triple combinations were between 0.2 and 0.5, indicated synergistic antitumor effect. To facilitate the drug combination study, the dose ratio of DPM, BEN and DEX was fixed to 100∶20∶1 (mass) and named DBDx.

**Table 1:** Antitumor activity of single agents, double and triple combinations in H22 tumor-bearing mice and the CI values.

Drugs, mg/kg			Fractional Inhibition, fa	CI‡
DPM	BEN	DEX		
Single agents				
100			0.228	
200			0.313	
	20		0.503	
	40		0.557	
		1	0.422	
		2	0.590	
		3	0.686	
Double combinations				
	40	2	0.663	0.976
100		2	0.647	0.845
200	40		0.587	0.838
Triple combinations				
100	40	2	0.800Δ **	0.402
200	40	2	0.827Δ **	0.339
300	40	2	0.874Δ **	0.231
100	20	2	0.792Δ	0.411
100	40	2	0.820Δ*	0.346
100	80	2	0.856Δ **	0.265
200	40	1	0.816Δ **	0.207
200	40	2	0.855Δ **	0.268
200	40	3	0.894Δ **	0.268

Δ P<0.01,compared with single agents.

**P<0.01, *P<0.05,compared with double combinations

‡ CI<1, CI = 1, and CI>1 indicate synergism, additivity, and antagonism, respectively.

### DBDx showed highly potent antitumor activity against human tumor xenografts

**Figure 1:**
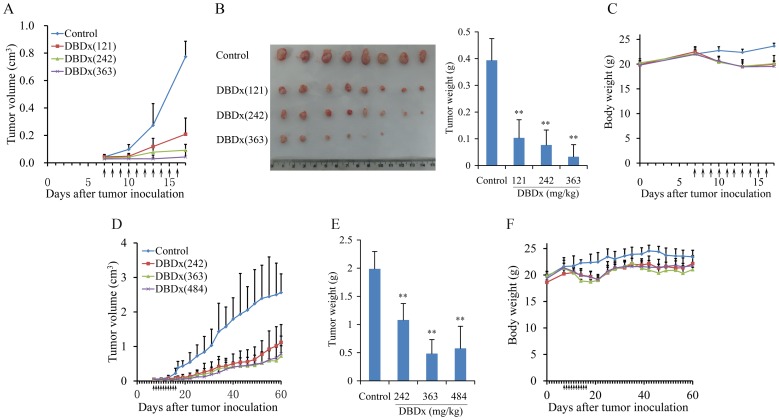
DBDx inhibited the growth of BEL-7402 xenograft in athymic mice. **A**. DBDx inhibited tumor growth in a dose-dependent manner. **B**. At day 17, mice were sacrificed. Tumors were photographed and tumor weights were measured. **C**. The body weights of mice during the 17 days' experiment. **D**. In the long term experiment, at day 60, namely 44 days after the final treatment, DBDx could still inhibit the tumor growth. **E**. At the end of the experiments, mice were sacrificed and tumor weights were measured. **F**. The animal body weights of all groups during the 60 day experiment. (Dose: mg/kg). Statistical significance was determined by Student's t-test; **P<0.01 compared with control.

The antitumor efficacy of DBDx was further evaluated in human hepatocellular carcinoma BEL-7402 xenograft model. After 7 days of tumor implantation, different doses of DBDx were given orally, once daily, for 10 days. Saline was given to mice of the control group. At day 17, DBDx at 121, 242 and 363 mg/kg inhibited tumor growth by 73.0%, 88.1% and 94.5% evaluated by tumor volume, respectively, statistically significantly different from that of the control group (P<0.01, Fig. 1.A). Notably, after dissection, it was found that the implanted tumor disappeared in 2 of 8 mice (Fig. 1.B).The tumor weights were shown in Fig. 1.B. During the experiments the body weight loss of the treated mice was less than 10% as compared with the body weight at the start of the experiment (Fig. 1.C). In another independent experiment, drugs were administrated as above, after 10 days of treatment the long-term antitumor effects of DBDx were evaluated. At day 60, namely 44 days after the last treatment, DBDx at 242, 363, and 484 mg/kg inhibited the tumor growth by 56.4%, 71.9% and 69.2% evaluated by tumor volume, respectively (Fig. 1.D), which was consistent with the tumor weight data (Fig. 1.E). The body weight of treated groups showed minor decrease (10%) during the treatment, while increased at the end of the experiments, indicating that the administered doses were well tolerated (Fig. 1.F).

**Figure 2:**
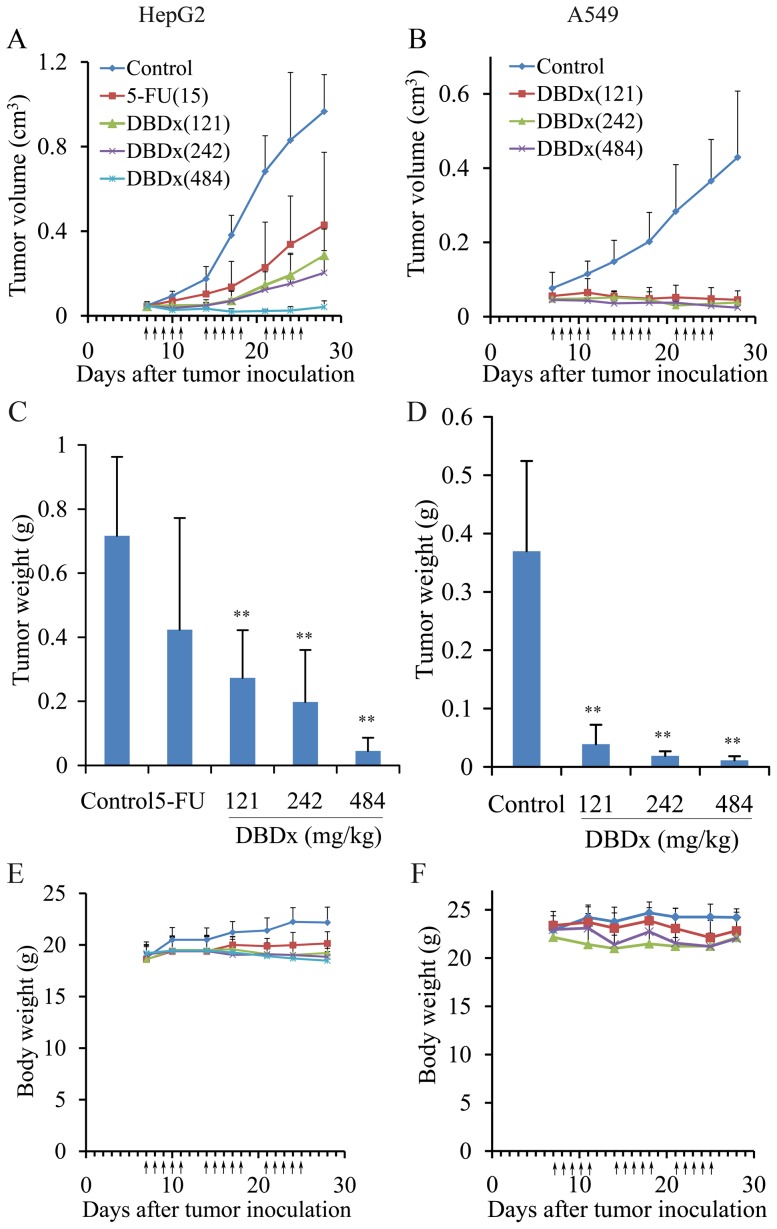
DBDx inhibited the growth of HepG2 xenograft and A549 xenograft in athymic mice. **A**,**C** and **E**. Tumor growth curve, tumor weight at the end of the experiment and the body weights of mice of all groups in HepG2 xenograft model. **B**, **D** and **F**. Tumor growth curve, tumor weight at the end of the experiment and the body weights of mice of all groups in A549 xenograft model. (Dose: mg/kg). Statistical significance was determined by Student's t-test; **P<0.01 compared with control.

In addition, the antitumor efficacy of DBDx was evaluated with other human cancer xenografts, including human hepatocellular carcinoma HepG2 and lung adenocarcinoma A549. Drugs were administrated orally, once daily, 5 consecutive days a week for 3 weeks (7–11 d, 14–18 d, 21–25 d). In HepG2 model, at day 28, DBDx at 121, 242 and 484 mg/kg inhibited tumor growth by 61.9%, 72.3% and 93.7% evaluated by tumor volume, respectively (Fig. 2. A). Meanwhile, 5-FU at 15 mg/kg inhibited tumor growth by 42%. The tumor weights of all groups were shown in Fig. 2.C. In A549 model, at day 28, DBDx at 121, 242 and 484 mg/kg inhibited tumor growth by 89.5%, 94.9% and 96.9% evaluated by tumor volume, respectively (Fig. 2.B). The tumor weights were shown in Fig. 2.D. Evidently, treated animals tolerated well to all above-mentioned doses of DBDx. As shown in Fig. 2.E and F, at the end of the experiment, no deaths occurred, and the body weight loss was less than 10% in all treated mice of various dosage groups, indicating that the treated animals tolerated well with the administered doses of DBDx.

### Comparison of antitumor efficacy of DBDx with other chemotherapy drugs

**Figure 3:**
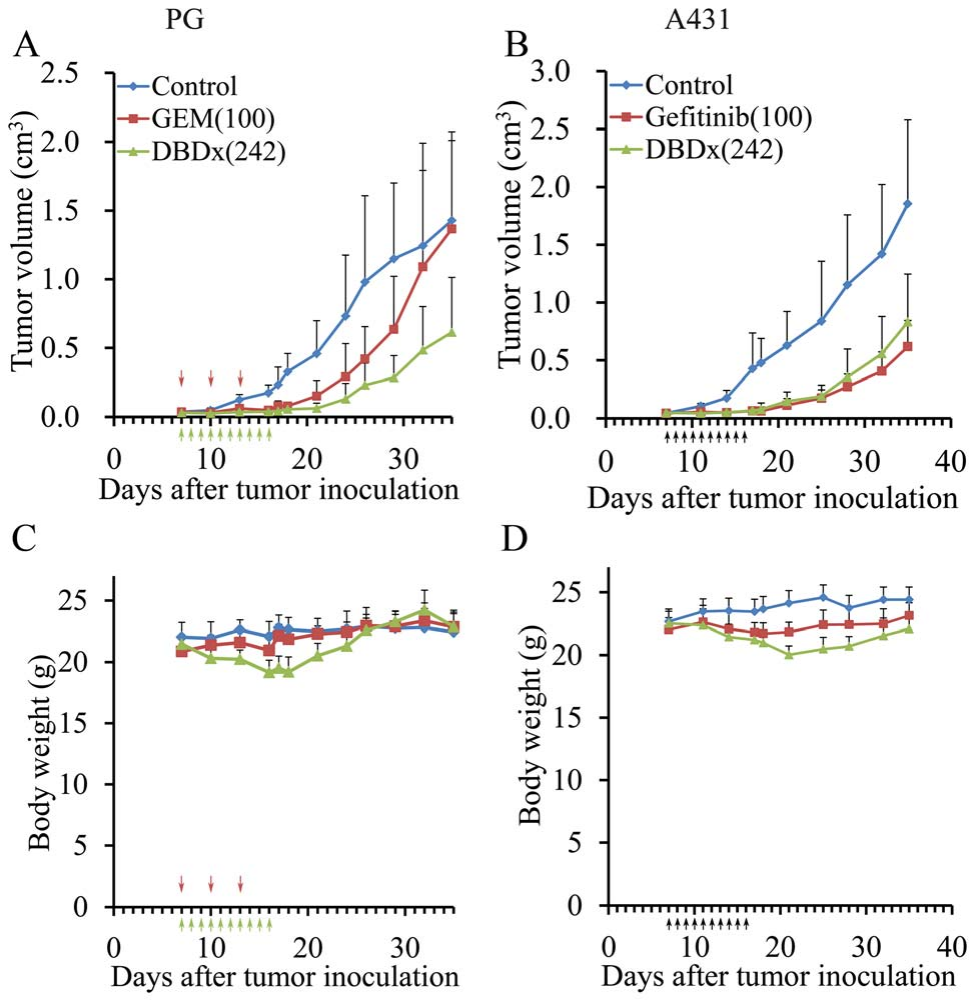
Comparison of antitumor efficacy of DBDx with GEM in PG xenograft model and gefitinib in A431 xenograft model. **A**. DBDX showed stronger antitumor activity than GEM in PG xenograft model. **B**. DBDX showed similar antitumor activity with gefitinib in A431 xenograft model. **C**. The body weights of PG xenograft-bearing mice during treatment. **D**. The body weights of A431 xenograft-bearing mice during treatment. (Dose: mg/kg).

The antitumor effect of DBDx was further compared with gemcitabine (GEM) and gefitinib. As well known, GEM, an antimetabolite drug, is commonly used in clinics for the treatment of lung cancer, pancreatic cancer, etc. In human lung carcinoma PG xenograft model, the antitumor activity of DBDx was compared with that of GEM. Seven days after tumor implantation, DBDx was given orally, once daily, for 10 days. GEM was given i.p. at day 7, 10 and 13, a total of three doses. As evaluated with tumor volume at day 17, GEM inhibited the tumor growth by 65.2%, while DBDx inhibited tumor growth by 82.6% which was significantly different from that of GEM (P<0.05, Fig. 3.A). At day 35, DBDx still exerted inhibition of tumor growth by 57.1%.

Gefitinib is an EGFR tyrosine kinase inhibitor. For comparative study, the efficacy of DBDx and gefitinib was evaluated with the human epidermoid carcinoma A431 xenograft model in which EGFR is highly-expressed. DBDx and gefitinib were respectively given orally, once daily, for 10 days. As shown in Fig. 3B, DBDx showed similar antitumor activity with gefitinib. At day 17, the inhibition rate for DBDx at 242 mg/kg was 84.3%, while gefitinib at 100 mg/kg inhibited tumor growth by 84.8%, respectively.

Both in PG and A431 models, the body weight loss of treated groups was less than 11%, and the body weight of treated mice in all groups increased at the end of the experiment, indicating that the treated animals tolerated well with administered doses of DBDx and gefitinib (Fig. 3.C, D).

### Toxicopathological examination

**Figure 4:**
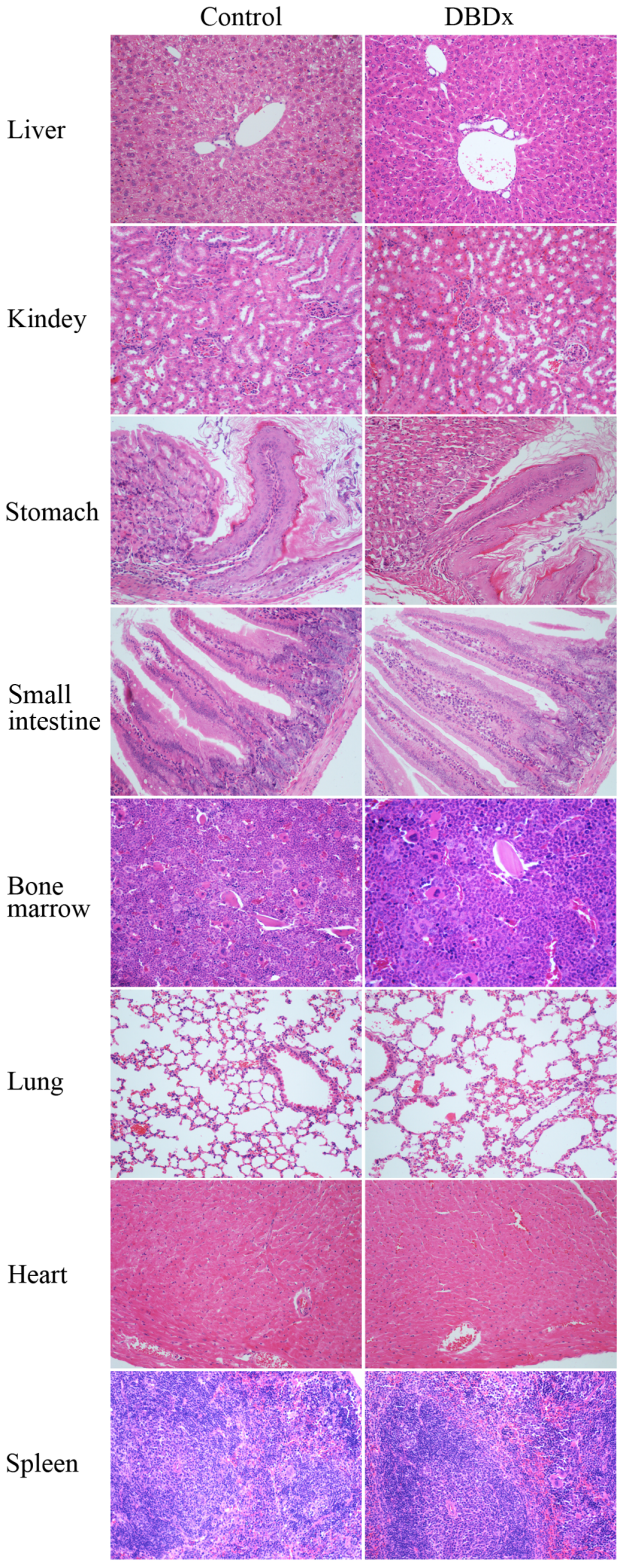
Toxicological studies in mice. Histopathological examination in HepG2 xenograft-bearing mice of the control group and the group treated with DBDx. No pathological changes were found in liver, kidney, stomach, intestine, bone marrow, lung, heart and spleen in DBDx-treated animals (H&E). (×200).

In HepG2 xenograft model described above, at day 28, DBDx at 242 mg/kg inhibited the tumor growth by 72.3%, histopathological examination (sections stained with H & E) showed no evidence of toxicological damage in liver, kidney, stomach, small intestine, bone marrow, lung, heart, and spleen (Fig. 4).

In H22 model, tumor-bearing Kunming mice were treated with DBDx at 242 mg/kg, once daily for 10 days. After 24 h of the last administration, histological sections of bone marrow were examined. Counts of the nucleated cells and the megakaryocytes in bone marrow showed no difference among normal mice, tumor-bearing mice treated with saline or DBDx (Table 2).

**Table 2:** Relative number of the nucleated cells and the megakaryocytes in femur bone marrow #.

Group	Tumor inhibition rate	Bone marrow nucleated cell (%)##	Bone marrow Megakaryocyte (%)
Normal mice	-	52.76	7.00
Tumor-bearing mice	-	62.31	6.28
Tumor-bearing mice treated with DBDx*	85.5%	52.33	7.54

# Relative number was determined by morphological analysis.

## Nucleated cells including the cells of myeloid and erythroid series.

*Mice treated with DBDx at 242 mg/kg, po, once daily for 10 days.

The acute toxicity of DBDx (per os) was examined in healthy Kunming mice. At the end of the experiment no animal deaths were observed, and the animal body weight increased to 28–38 g even when the dose of DBDx went up to 2 000 mg/kg, showing a good safety *in vivo*. Fur change and behavior abnormality were not observed.

### In vitro cytotoxicity assay of DBDx to cultured tumor cells

The cytotoxicity of DBDx and single agents was evaluated in vitro using clonogenic assay. Bestatin and dexamethasone showed weak cytotoxicity to cultured BEL-7402 cells. The IC_50_ values of them were above 100 µg/mL. For dipyridamole and DBDx, the IC_50_ values were 13.34±0.65 and 17.48±0.59 µg/mL, respectively.

### Antitumor mechanism studies of DBDx

**Figure 5:**
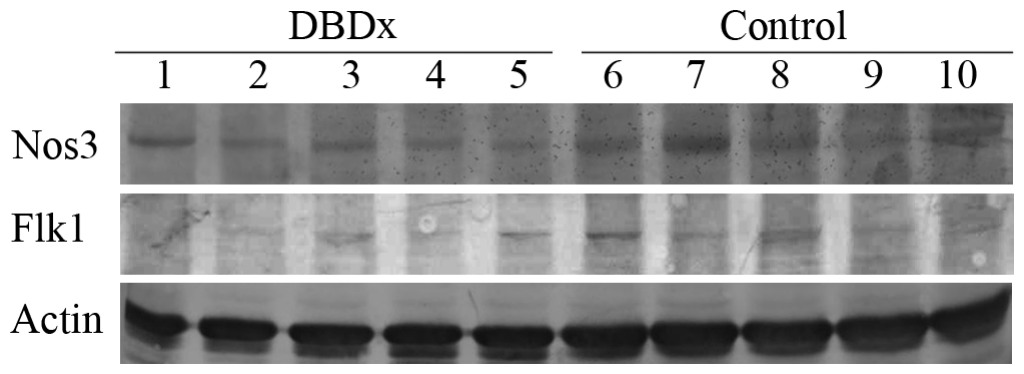
DBDx treatment down-regulated the expression of Nos3 and Flk1. Five tumor tissue specimens taken from each group of hepatoma 22 (H22) transplanted Kunming mice were used.

The changes of protein expression in transplanted hepatoma 22 after DBDx treatment were investigated. We analyzed a series of growth factors including epidermal growth factor (EGF), vascular endothelial growth factor (VEGF), transforming growth factor beta (TGF-β) and fibroblast growth factor 2 (FGF2); growth factor receptors such as EGFR (EGF receptor), Flk1 (VEGF receptor 2); proteins related to tumor cell survival and apoptosis such as Bcl-2 and survivin; and some other proteins such as Nos3. As a result, two proteins Flk1 and Nos3 were found to be down-regulated in the DBDX-treated group (Fig. 5).

**Figure 6:**
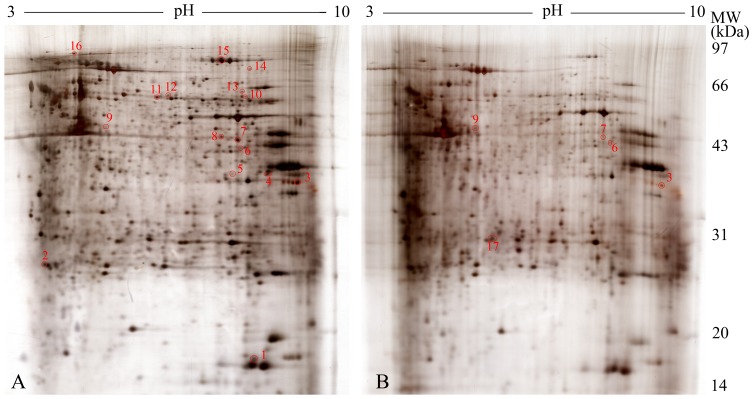
A representative protein profile obtained from Bel-7402 xenograft treated with saline or DBDx. After separation on 17 cm, pH 3–10 linear strips in the first dimension and on a 12% SDS-PAGE in the second dimension, proteins were stained with silver. **A**. Saline-treated group. **B**. DBDx-treated group. Protein spots marked on the maps were excised from the gels and sequentially identified by MS.

Next, the global protein difference between the saline control and the DBDx-treated groups was compared by 2-DE and MS. Tumor tissues from human hepatocarcinoma BEL-7402 xenograft in athymic mice were used. About 700 protein spots were displayed per gel. Fig. 6 showed the representative protein profile obtained from 2-DE. Statistical analysis of the normalized volume of matched spots revealed 33 protein spots whose intensity showed>3-fold difference between saline and DBDX-treated groups. From these protein spots, 17 differentially expressed proteins were identified (Table 3). Among these identifications, 12 were unique in two groups, 4 down-regulated and 1 up-regulated in DBDx-treated group. Other relevant information listed in Table 3 included the NCBI accession numbers, fold changed, mascot score, theoretical and experimental molecular weight, theoretical and experimental pI and sequence coverage.

**Table 3:** List of differentially expressed proteins from Bel-7402 xenograft-bearing mice treated with DBDx and saline as identified by MALDI-TOF-TOF analysis.

Spot no^a^	Accession no^b^	Description	Fold changed^c^	Mascot score^d^	MW (kDa) the./exp.^e^	pI the./exp.^f^	Sequence coverage
1	gi|1633054	Chain A, Cyclophilin A Complexed With Dipeptide Gly-Pro	-	147	18.1/16.7	7.82/8.58	69%
2	gi|33285832	TCTP	-	79	19.7/26.3	4.98/3.52	38%
3	gi|4504447	heterogeneous nuclear ribonucleoprotein A2/B1 isoform A2	-3.14	235	36.0/36.7	8.67/9.62	56%
4	gi|31645	glyceraldehyde-3-phosphate dehydrogenase	-	92	36.2/37.6	8.26/8.88	39%
5	gi|4505823	pirin	-	139	32.2/37.8	6.42/8.04	45%
6	gi|157834561	Chain A, Aldehyde Reductase	-4.55	80	36.8/41.5	6.34/8.36	24%
7	gi|67464043	Chain O, Crsytal Structure Of Human Liver Gapdh	-6.67	104	36.5/42.6	8.58/8.17	29%
8	gi|460771	hnRNP-E1	-	150	38.0/42.7	6.66/7.94	41%
9	gi|4507215	signal recognition particle 54 kDa isoform 1	6.48	67	56.0/46.5	8.87/5.00	25%
10	gi|67464392	Chain A, Structure Of Human Muscle Pyruvate Kinase (Pkm2)	-	99	60.3/56.8	8.22/8.4	38%
11	gi|5453603	chaperonin containing TCP1, subunit 2	-	265	57.8/57.5	6.01/6.23	63%
12	gi|196049886	Chain A, Crystal Structure Of Human 3-Oxoacid Coa Transferase 1	-	107	53.3/58.3	5.89/6.50	32%
13	gi|169404695	Chain A, Pyruvate Kinase M2 Is A Phosphotyrosine Binding Protein	-	76	57.1/59.8	8.00/8.29	22%
14	gi|16878077	FUBP1	-	147	68.8/73.0	6.85/8.47	25%
15	gi|3192917	inducible nitric oxide synthase	-5.15	77	132.6/80.5	8.11/7.79	18%
16	gi|16507237	heat shock 70 kDa protein 5	-	285	72.4/87.2	5.07/4.23	44%
17	gi|662841	heat shock protein 27	+	125	22.4/29.7	7.83/5.55	48%

a Spot number referred to Fig. 7.

b Accession number for the identification in NCBI database.

c Positive and negative fold change indicates up- and down-regulation of protein expression compared with control, respectively. Minus without value means the protein only appeared in control group, and plus means the opposite.

d Protein scores greater than 66 are significant (p<0.05)

e Theoretical (the.) and experimental (exp.) molecular weight of the matching protein in kDa.

f Theoretical (the.) and experimental (exp.) isoelectric point of the matching protein.

Then these identified proteins were categorized according to biological process and signal pathway using PANTHER classification system. Analysis of the biological process showed that metabolic process (40.6%) and immune system process (12.5%) were mainly affected by DBDx treatment. Other biological process affected by DBDx included cell communication, cellular process, response to stimulus, system process, transport and apoptosis and generation of precursor metabolites and energy (Fig. 7A). Analysis of the cellular signaling pathways showed that angiogenesis, VEGF signaling pathway and glycolysis encompassed 42.9% of the signaling pathways affected by DBDx treatment (Fig. 7B). Other affected pathways included apoptosis signaling pathway, endothelin signaling pathway, Huntington disease, interleukin signaling pathway, PI3 kinase pathway, Parkinson disease, pyruvate metabolism, and p38 MAPK pathway.

**Figure 7:**
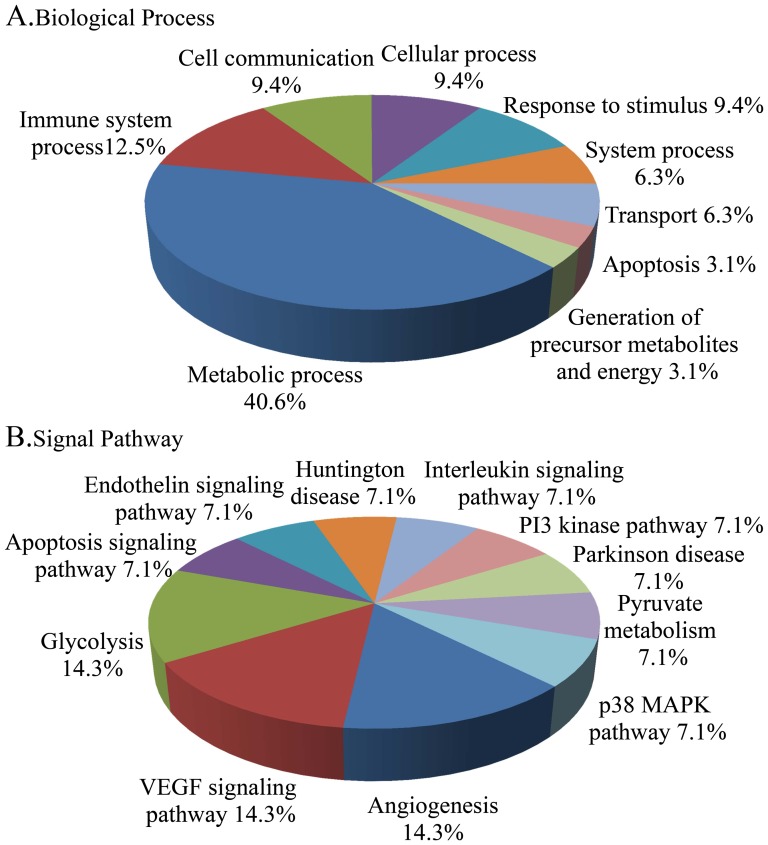
Analysis of the biological processes (A) and signal pathways (B) affected by DBDx treatment using PANTHER.

## Discussion

Drug combinations are widely used in cancer treatment for over half a century. It is a rational and efficient strategy to increase therapeutic efficacy, while decrease toxicity and overcome resistance. In the present study, we proposed a tumor microenvironment-oriented, multifunctional drug combination strategy that aims at a highly potent therapeutic efficacy *in vivo*. A triple drug combination that comprises dipyridamole, bestatin and dexamethasone was designed and its therapeutic effectiveness has been confirmed. DBDx, the triple drug combination, is unique for comprising low-cytotoxic agents other than conventional chemotherapeutics and for accomplishing the remarkable therapeutic efficacy at well tolerated dosage levels. In general, examination of therapeutic efficacy *in vivo* with experimental tumor systems, especially human cancer xenografts in athymic mice, is of particular importance for the evaluation of anticancer agents. As shown, DBDx is highly effective against the growth of human hepatocellular carcinoma BEL-7402 xenograft. Besides reducing tumor size markedly, DBDx treatment even caused the implanted tumor entirely disappeared in 2 of 8 mice.

Notably, DBDx displayed a broad-spectrum antitumor activity. When the dose of DBDX reached 484 mg/kg, a tolerable dosage, the inhibition rates were up to 90% in all of the tested xenograft models, including human hepatocellular carcinoma HepG2, lung adenocarcinoma A549, lung giant cell carcinoma PG, and epidermoid carcinoma A431 xenografts. The broad-spectrum antitumor activity implies that DBDx might be acting mainly via modulating the tumor microenvironment; consequently, it is relatively independent of tumor type. Furthermore, the combination of 3 drugs with different mechanisms of action may also provide multi-modal coverage of a broad spectrum of tumors.

For drug combinations, the therapeutic activity is not only dependent on the dose intensity but also on the dose ratios of the combined components. Some ratios of combined drugs can be synergistic, while other ratios of the same agents may be additive or even antagonistic [20]. In all of our tested combinations, the three agents acted synergistically (CI: 0.2–0.5). As known, synergistic combination may increase the therapeutic efficacy at tolerated doses and slow down the development of drug resistance [21]. It is of interest that the three agents of DBDx combination are not conventional cytotoxic chemotherapeutics; by contrast, they mainly affect the tumor microenvironment. Therefore, this multifunctional drug combination might be capable of reducing the emergence of drug resistance.

Though DBDx shows highly potent antitumor efficacy *in vivo*, clonogenic assay demonstrates that this combination does not display potent cytotoxicity *in vitro*. It indicates that the inhibition of tumor growth by DBDx *in vivo* does not mainly rely on the killing of tumor cells directly; by contrast, DBDx might exert its antitumor activity predominantly through interfering with the tumor microenvironment. As reported, dipyridamole is an active inhibitor of nucleoside transport. Bestatin can target to aminopeptidase N and inhibit tumor angiogenesis [15]. Dexamethasone can also suppress angiogenesis [17]. Our mechanistic studies proved that DBDx down-regulated the expression of Flk1, a receptor for VEGF, and NOS3, which can regulate vascular function [22]. Besides acting on tumor angiogenesis, DBDx also affects the immune system and inflammation. As reported, inflammation plays an important role in tumorigenesis and progress [23], [24]. Bestatin is a widely used immunomodulator against tumor. Dexamethasone is an anti-inflammatory drug. A recent report shows that dipyridamole significantly decreases the immune cell infiltration and serum inflammatory cytokines levels in mice [9]. The effects of DBDx on tumor microenvironment were further proved by 2-DE and following PANTHER analysis. Classification of differentially expressed proteins by the cellular signaling pathways revealed that DBDx mainly affected the angiogenesis and VEGF signaling pathway. Analysis of the biological process showed that immune system process was affected. These results imply that DBDx exerts its antitumor activity predominantly through interfering with the tumor microenvironment. The effects of DBDx on tumor angiogenesis and immue system will be further investigated in animal models.

## Conclusions

As a whole, DBDx, the three drug combination, shows highly remarkable antitumor efficacy *in vivo*. Because of its low-cytotoxic attributes and its broad-spectrum antitumor activity, this multifunctional combination might be useful in the treatment of cancers refractory to conventional chemotherapeutics. Moreover, this combination may further integrate with various chemotherapeutics to constitute new highly effective regimens. Based on its mechanism, DBDx might also provide hopes for treatment of multidrug-resistant or relapsed cancers.
